# FAST: A Novel, Executive Function-Based Approach to Cognitive Enhancement

**DOI:** 10.3389/fnhum.2019.00235

**Published:** 2019-08-02

**Authors:** Jessamy Norton-Ford Almquist, Santosh Mathan, Anna-Katharine Brem, Franziska Plessow, James McKanna, Emiliano Santarnecchi, Alvaro Pascual-Leone, Roi Cohen Kadosh, Misha Pavel, Nick Yeung

**Affiliations:** ^1^Honeywell Labs, Honeywell Aerospace, Redmond, WA, United States; ^2^Department of Neurology, Berenson-Allen Center for Non-Invasive Brain Stimulation, Beth Israel Deaconess Medical Center, Division for Cognitive Neurology, Harvard Medical School, Boston, MA, United States; ^3^Department of Experimental Psychology, Medical Sciences Division, University of Oxford, Oxford, United Kingdom; ^4^Department of Electrical and Computer Engineering, Northeastern University, Boston, MA, United States

**Keywords:** cognitive training, cognitive enhancement, fluid intelligence, executive function, transcranial electrical stimulation (tES), FAST

## Abstract

The present study introduces a novel cognitive intervention aimed at improving fluid intelligence (G*f*), based on a framework we refer to as FAST: Flexible, Adaptive, Synergistic Training. FAST leverages a combination of novel game-based executive function (EF) training—designed specifically to enhance the likelihood of transfer—and transcranial electrical stimulation (tES), with aims to synergistically activate and strengthen mechanisms of cognitive control critical to G*f*. To test our intervention, we collected three G*f* measures from 113 participants [the advanced short Bochumer Matrizen-Test (BOMAT), Raven’s Advanced Progressive Matrices (APM), and matrices similar to Raven’s generated by Sandia labs], prior to and following one of three interventions: (1) the FAST + tRNS intervention, a combination of 30 min of daily training with our novel training game, Robot Factory, and 20 min of concurrent transcranial random noise stimulation applied to bilateral dorsolateral prefrontal cortex (DLPFC); (2) an adaptively difficult Active Control intervention comprised of visuospatial tasks that specifically do not target G*f*; or (3) a no-contact control condition. Analyses of changes in a G*f* factor from pre- to post-test found numerical increases for the FAST + tRNS group compared to the two control conditions, with a 0.3 SD increase relative to Active Control (*p* = 0.07), and a 0.19 SD increase relative to a No-contact control condition (*p* = 0.26). This increase was found to be largely driven by significant differences in pre- and post-test G*f* as measured on the BOMAT test. Progression through the FAST training game (Robot Factory) was significantly correlated with changes in G*f*. This is in contrast with progress in the Active Control condition, as well as with changes in individual EFs during FAST training, which did not significantly correlate with changes in G*f*. Taken together, this research represents a useful step forward in providing new insights into, and new methods for studying, the nature of G*f* and its malleability. Though our results await replication and extension, they provide preliminary evidence that the crucial characteristic of G*f* may, in fact, be the ability to combine EFs rapidly and adaptively according to changing demand, and that G*f* may be susceptible to targeted training.

## Introduction

Fluid intelligence (G*f*) has been defined as the ability to flexibly apply one’s knowledge and skills to novel situations (Cattell, 1971; Carpenter et al., 1990). This ability is crucial in learning and adaptive behavior, and in navigating complex environments where one must maintain multiple competing goals (Gottfredson, 1997; Gray and Thompson, 2004; Deary et al., 2007). The question of whether focused training of specific cognitive skills can enhance G*f* is currently a topic of intense debate (Shipstead et al., 2012; Melby-Lervåg and Hulme, 2013, 2015; Au et al., 2014; Karbach and Verhaeghen, 2014; von Bastian and Oberauer, 2014), and is one of substantial practical and theoretical importance: G*f* is a strong predictor of academic success, lifetime earnings and other significant life outcomes (Gottfredson, 1997; Gray and Thompson, 2004; Deary et al., 2007), such that enhancement through training could confer valuable benefits. In terms of theory, identifying cognitive skills that can be trained to produce enhanced G*f* would provide valuable evidence regarding the core component processes of G*f*, which have proven elusive to date. With the stakes high, the literature is burgeoning with new training studies, including prominent examples of successful positive transfer from focused cognitive training with tests that load strongly on G*f* (Jaeggi et al., 2008, 2014; Rudebeck et al., 2012; Salminen et al., 2012), as well as prominent failures to replicate transfer (Owen et al., 2010; Chooi and Thompson, 2012; Harrison et al., 2013; Thompson et al., 2013).

Skills acquired through cognitive training will only transfer to novel domains if the training tasks share component processes with the broader skills targeted for enhancement, and if the learned task encodings are general enough to be applied to novel contexts (Singley and Anderson, 1985; VanLehn, 1996; Jaeggi et al., 2008; Shipstead et al., 2010; Taatgen, 2013). Many studies to date have taken the approach of using working memory training tasks to enhance G*f*, given that working memory has been proposed as a core executive function (EF) underlying G*f* (Wiley et al., 2011; Diamond, 2013). Similar brain regions are activated during performance of working memory tasks (Burgess et al., 2011) and tasks thought to depend on G*f* (Halford et al., 2007). Additionally, measures of working memory capacity have been shown to explain at least half of variance in G*f* across individuals (Kane et al., 2005).

However, evidence of positive transfer from working memory training to G*f* task performance remains mixed, with some analyses finding evidence of significant near and far-transfer (Schmiedek et al., 2010; Au et al., 2014; Karbach and Verhaeghen, 2014), and others finding limited or no transfer effects (Melby-Lervåg and Hulme, 2013; Rapport et al., 2013; Lampit et al., 2014). Inconsistent findings, combined with methodological issues such as lack of active control groups and small sample sizes, have complicated the larger picture of its effectiveness. Many studies to-date vary task difficulty while holding the task and stimuli fixed over training (e.g., strategy training approaches, reviewed in Verhaeghen et al., 2004; Jaeggi et al., 2008; Morrison and Chein, 2011), leading to repetitive practice that previous work on skill acquisition suggests may produce task-specific strategies rather than general skills that should transfer across contexts (Chase and Ericsson, 1982). Furthermore, studies have often lacked an appropriate active control condition (Shipstead et al., 2010; Dougherty et al., 2016), finding improvements only in comparison to no-contact controls and allowing for the possibility that Hawthorne or other placebo-like effects are the true source of improvement following training.

Considered together, prior research does not offer a consistent answer to the question of whether working memory training can enhance G*f*. But, prior research does point to avenues for enhancing the effects of cognitive training on G*f*. The first opportunity for improvement comes from EF research suggesting that a broader set of EF processes, besides working memory, contribute to G*f*. As detailed in Diamond (2013), G*f* represents a set of higher-order reasoning and problem-solving EFs, which are influenced by and share neural resources with three core EFs: working memory, cognitive flexibility and inhibition (Duncan and Owen, 2000; Duncan et al., 2012). These functions have been characterized as the core components of cognitive control, and the building-blocks of complex adaptive behaviors (Miyake et al., 2000; Friedman et al., 2008; Collins and Koechlin, 2012; Lunt et al., 2012). Therefore, rather than focusing on working memory training alone, all three core EFs should be considered. The second avenue for improving G*f*-related cognitive training comes from the learning transfer literature, suggesting that the ability to encode generalizable knowledge crucially depends on the diversity of contexts in which skills are acquired and practiced.

These gaps have informed the FAST (Flexible, Adaptive, Synergistic Training) training framework. The FAST approach reflects a hypothesis that a training environment that supports broad contextual variation and broadly emphasizes all core EF processes will tap into networks relevant to G*f* and will foster more effective skill development. The FAST approach is designed to engage all three core EFs [working memory, cognitive flexibility, and inhibitory control (Monsell, 1996; Miyake et al., 2000; Diamond, 2013)], and do so in various combinations across a rich variety of environments. EFs are exercised through variants of well-established task-types, including for working memory the *n*-back task (Verhaeghen et al., 2004; Owen et al., 2005; Verhaeghen and Basak, 2005), for cognitive flexibility the task switching paradigm (Rogers and Monsell, 1995), and for inhibition the go/no-go (Donders, 1969) and stop-signal tasks (Logan and Cowan, 1984).

A second empirically-grounded hypothesis of FAST is that the combination of the training with noninvasive brain stimulation (NIBS) may synergistically boost the activity of cortical areas thought to be critical to G*f*, and promote modulations in neural activity (e.g., changes in cortical excitability, network connectivity, plasticity and/or increased signal-to-noise ratios) during training that can lead to enhanced cognitive function in healthy individuals (Krause and Cohen Kadosh, 2013; Cohen Kadosh, 2015; Santarnecchi et al., 2015; Looi et al., 2016). In this initial test of the combination of FAST and NIBS, we used transcranial random noise stimulation (tRNS). Researchers have suggested that tRNS can increase cortical excitability through mechanisms of stochastic resonance (Terney et al., 2008), which, combined with the appropriate task, can increase learning (Fertonani et al., 2011; Santarnecchi et al., 2015). Recent results have highlighted the particular benefits of tRNS applied to bilateral dorsolateral prefrontal cortex (DLPFC) in the acquisition and retention of high-level cognitive skill (Snowball et al., 2013), and the effect of tRNS has been shown to be more effective as task difficulty increases (Popescu et al., 2016), which may be beneficial in a training context. Work to-date exploring the impact of alternative stimulation approaches to enhancement of G*f* has seen mixed results, with recent studies of transcranial alternating current in theta band (Pahor and Jaušovec, 2014) and gamma band frequencies (Santarnecchi et al., 2013) seeing positive offline results in terms of performance enhancement on G*f* tests like Raven’s Advanced Progressive Matrices (APM), and a recent examination of the impact of transcranial direct current stimulation finding detrimental offline effects of stimulation on metrics of the Weschler adult intelligence scale IV (Sellers et al., 2015).

In light of the substantive methodological issues in the field reviewed above, and the considerable theoretical uncertainty about whether it is possible at all to enhance G*f* relative to appropriately strict controls, we focused here on the combined intervention of FAST+tRNS, which we theorized would have the greatest likely efficacy. This approach left open—for the moment—the question of whether FAST, tRNS, or the combination thereof would critically underpin any observed effects. Thus, this study aimed to assess whether a compound intervention, consisting of a combination of FAST+tRNS, could lead to enhancement of G*f* relative to both a no-contact and active control condition.

Besides augmenting G*f* training as noted above, we address methodological weaknesses that have been noted in working memory training research. Recent work has highlighted the need for comparison of cognitive interventions against active control conditions (Shipstead et al., 2012). We assessed the impact of the FAST+tRNS training across 9–11 daily sessions *via* changes in performance from pre- to post-training on a suite of established tasks that load strongly on G*f*, and contrasted these results with those from an active control training of equivalent duration, as well as a no-contact control condition. The comparison of our intervention against an active control condition is particularly important, given recent prominent findings of null results in the literature when such a comparison is made (Chooi and Thompson, 2012; Redick et al., 2013). We also collected multiple measures of G*f* in keeping with recommended best practices for determining the generalizability of results (Shipstead et al., 2010). With several measures, shared (non-task specific) variance can be established by means of factor analysis or composite score (Kim and Mueller, 1978). The active control condition was administered similarly to the FAST training and was designed to maintain participant motivation by including several tasks that were adaptive in their difficulty based on participant performance. Crucially, the active control training differed from FAST by targeting lower-level perceptual abilities, rather than high-level EFs. Additionally, a third group of participants served as passive (no-contact) controls, performing on the pre- and post-test measures with no intervening training.

## Materials and Methods

### Participants

A total of 113 participants across three data collection sites gave their informed consent to participate in the study: Beth Israel Deaconess Medical Center (BIDMC; *n* = 36), University of Oxford (*n* = 36) and Northeastern University (NEU; *n* = 41). Participant exclusion criteria included a history of health problems such as epilepsy, migraines, neurological and psychiatric disorders. Participants were required to have normal or corrected vision and hearing, and agree to abstain from alcohol throughout the study, and refrain from caffeine consumption within 2 h of daily training. Of the 113 participants from whom data was collected, 14 participants were excluded from the analysis due to either an error in the progression of FAST that stopped participants from reaching the highest level of our training (*n* = 10), or due to non-compliance with the test administration on at least two independent measures (*n* = 4). In addition to these 14, the initial seven participants of the study were administered a shorter version of the Bochumer Matrizen-Test (BOMAT), which had been used in a pilot study, and one participant was found to have been administered the BOMAT post-test at both the pre- and post-test sessions. Data from these participants were also excluded, and as a result final analyses consisted of pre-test and post-test performance for 91 participants across the three conditions (FAST+tRNS: *n* = 32, age 22.4 ± 3.4; active control: *n* = 30, age 24.57 ± 4.54; No-Contact: *n* = 29, age 23.4 ± 4.3; Harvard BIDMC: *n* = 27; NEU: *n* = 31; Oxford: *n* = 33).

Participants in the study were pseudorandomly assigned to one of three conditions: no-contact control (NC), active control (AC), or training (FAST+tRNS), such that group sizes and baseline characteristics were balanced as well as possible, One-way analysis of variance of the three groups found no significant differences in age (NC: *μ*= 23.38 years, SD = 4.25; AC: *μ* = 25.57, SD = 4.54; FAST+tRNS: *μ* = 22.4, SD = 3.42), years of education (NC: *μ* = 16.93, SD = 2.68; AC: *μ* = 17.23, SD = 3.13; FAST+tRNS: *μ* = 16.03, SD = 2.48), or G*f* at pre-test (NC: *μ* = 0.09, SD = 1.09; AC: *μ* = 0.07, SD = 1.06; FAST+tRNS: *μ* = −0.14, SD = 0.87).

In the US, these studies were approved by the institutional review board at all participating institutions (Harvard BIDMC: Committee on Clinical Investigations/IRB, Protocol 2014P-000024; Northeastern: Human Subject Research Protection/IRB, Protocol 14-08-15) and in the UK by the Berkshire National Research Ethics Committee (REC reference 14/SC/0131). All participants gave written informed consent prior to training, according to the Declaration of Helsinki, and were remunerated for their participation (£12 and $11–20 per hour depending on site, in the UK and the US, respectively).

### A Novel Approach: FAST

A fundamental aspect of the FAST framework is that it is designed to foster the development of general skill encoding through practice in highly variable contexts, in order to support transfer. This crucial flexibility of FAST comes from the wide array of tasks it includes, based on the use of unique (factorial) combinations of EFs, as well as the inclusion of an array of other critical cognitive elements throughout. For example, FAST tasks engage several domains of working memory through the use of different stimulus types and task contexts (visual: pictorial and spatial; symbolic: verbal and numeric). FAST also utilizes logical and relational cognitive operators in many of the tasks; for example some tasks require that participants sort stimuli based on whether they are both a certain color and shape (logical: and), or based on whether they come from the same semantic category (e.g., “animal"; relational: semantic). In the highest levels of training, FAST also includes tasks that require hypothesis-testing, where flexible task-model construction must occur.

Finally, a crucial feature of FAST is the requirement for participants to engage in rapid instructed task learning (Cole et al., 2013). The ability to generate new task models flexibly and reliably in response to changing instructions has been proposed to be central to the relationship between working memory and G*f* (Salthouse and Pink, 2008) and could also be a crucial factor in the relationship of G*f* with cognitive flexibility and inhibitory control. In particular, working memory tasks that involve instructed performance of this kind have been shown to very strongly activate the network of brain regions implicated in high-level reasoning and problem solving (Dumontheil et al., 2011) and correlate more strongly with G*f* than standard working memory paradigms (Duncan et al., 2012). All components of FAST require participants to rapidly encode novel sets of instructions and use these to guide their action. This encode-perform cycle is repeated with entirely new task requirements every 2–3 min, with little or no repetition of specific tasks within or between training sessions, such that FAST emphasizes the general capacity to flexibly configure cognitive resources according to current requirements, rather than the specific ability to adopt any one configuration in particular.

Although FAST incorporates a very large task space, the particular progression of tasks encountered by a participant is adaptively controlled by measures of success, as well as an underlying structure of task “levels". Level 1 tasks involve only one EF (single-EF tasks), such as switching between categorizing object pictures as animal/non-animal or flying/non-flying, depending on the color of a light that appears with the stimulus (cognitive flexibility). Level 2 tasks combine two EFs (dual-EF tasks), such as tasks asking participants to both switch between categorizing object pictures as animal/non-animal or flying/non-flying depending on a cue light, and to withhold responses (inhibition) when a no-go stimulus cue is present (e.g., a yellow “stop” signal). Level 3 tasks combine one or two EF components with a cognitive operator of logical or relational contrasts, such as switching between categorizing object pictures as animal/non-animal or flying/non-flying, depending on whether the yellow “stop" signal or a cue light appears (not both; logical xOR). Level 4 tasks come in two varieties. Some tasks combine all three core EFs, such as switching between categorizations while withholding responses if the current stimulus matches the stimulus presented three trials previously (working memory). These Level 4 tasks incorporate an imbalance among the EF sub-components, such that a dual-EF task is performed for the majority of trials (primary task), and in the case of an intermittent cue, a single-EF task is performed (secondary task). This imbalance among the EF sub-tasks serves as a means of training against goal neglect (Duncan and Owen, 2000), a key component of G*f* which, to our knowledge, has rarely if ever been explicitly incorporated into a training regimen. In addition, certain of these Level 4 tasks incorporate a dual n-back component, which has been tied to instances of transfer to improvement in G*f* in well-known previous work (Jaeggi et al., 2008). Finally, the second type of Level 4 tasks requires the participant to engage in hypothesis-testing in order to infer the appropriate tasks to perform, such as inferring the correct categorization rule for object pictures based on the color of a light that appears with the stimulus, without explicit instruction to do so.

With the increasing number of components present in the levels of our game, we are able to iteratively expose participants to increasingly complex and diverse environments of EF practice. For example, a participant will encounter tasks targeting inhibition alone (Level 1 tasks) before they encounter inhibition tasks that also include either working memory (inhibition + working memory, Level 2) or cognitive flexibility (inhibition + cognitive flexibility, Level 2). All of this will precede tasks that also include a logical or relational operator (e.g., inhibition + working memory + xOR, Level 3), which are then followed by either those that include all three EFs (inhibition + working memory + cognitive flexibility, Level 4a), or those that include hypothesis-testing (Level 4b). While we predicted that our four levels would roughly contain tasks of increasing difficulty (e.g., Level 3 tasks would be harder than Level 2 tasks), tasks were further ordered according to relative difficulty, based on participant performance in previous pilot data. We used the resulting order to move participants through tasks of increasing difficulty (roughly sequentially through our four task levels), while maintaining approximately equal exposure to the three core EFs at all times. We also prioritized advancing participants into Level 2+ tasks fairly quickly, so participants spent the least amount of time on Level 1 (single-EF) tasks.

### Procedure

Our study included three groups, to which participants were randomly assigned according to a stratified randomization process based on age, education, and gender. These groups included: individuals who received the FAST+tRNS intervention, individuals in an active control condition (AC), and individuals in a no-contact control condition (NC). Participants in the FAST+tRNS and active control groups were enrolled in the study for 13 days across 3 weeks, including an initial day of pre-tests, 9–11 days of training, and 1 day of post-tests. Participants were allowed to miss no more than two (total) days of training and remain enrolled in the study (minimum number of training days = 9). Participants were asked to refrain from drinking alcoholic beverages in the evenings before training sessions, and from drinking caffeinated beverages within 1 h of training. Participants were also instructed that compliance with the study protocol required at least 6 h of sleep each night during enrollment in the study. However, in this study sleep monitoring was not included.

Pre- and post-tests consisted of three tests of G*f* as detailed below, and were completed in a single session lasting approximately 1 h. Parallel versions of the G*f* tests were administered at the pre- and post-test sessions. On training days, participants in the FAST+tRNS and active control groups engaged in their respective training interventions for 30 min per day, during which time they engaged in a variety of either cognitive (FAST+tRNS) or visuospatial tasks (active control). In the case of the FAST+tRNS group, the final 9 days of training included 20 min of transcranial random noise stimulation (tRNS), applied bilaterally over dorsal prefrontal regions (days one and two had no stimulation). The onset of stimulation was aligned with the start of the training and included a 30-s ramp-up and ramp-down. Stimulation current (1 mA) was delivered *via* pi-electrodes (3.14 cm^2^) to channels F3 and F4 of the International 10-20 EEG system. Participants were informed of the possibility that they might receive stimulation during informed consent, and all participants were blinded as to the existence of multiple forms of training (AC and FAST+tRNS).

The total duration of each training session for the FAST+tRNS and active control groups was approximately 1 h, with 30 min of intervention, several minutes for breaks, and approximately 20 min for equipment set-up and paperwork. No-contact Control participants completed all pre-test and post-tests on the same schedule as the FAST+tRNS and active control groups, but received no training in-between.

### Apparatus

All tests and training were administered on DELL PCs equipped with the Windows 7 operating system. All G*f* tests (BOMAT, Raven’s, and Sandia) and active control tasks were implemented with Presentation software (Version 17.0; Neurobehavioral Systems, Inc., Albany, CA, USA). Transcranial electrical stimulation (tES) was applied using the Starstim™ system developed by Neuroelectrics (Barcelona, Spain). The Starstim™ wireless system offers flexible placement of up to eight electrodes according to the 10-20 system. Each electrode can be configured for either stimulation or recording, allowing for simultaneous delivery of electrical stimulation and EEG data acquisition. All EEG activity was recorded in real-time *via* an application programming interface developed by Neuroelectrics—analyses of the EEG data are not reported here.

### Brain Stimulation

The stimulation protocol used in this study involved transcranial random noise stimulation (tRNS) over DLPFC (channels F3 and F4). While there is some debate as to whether the neurobiological substrate of intelligence is related to activity of a diffuse network vs. a restricted number of brain regions (Jung and Haier, 2007), the nature of the brain stimulation we were able to apply in the present investigation posed some constraints in terms of the number and location of available target regions (i.e., only two electrodes could be placed on the scalp). We, therefore, informed our decision of target location with results of previous studies of tES over DLPFC, which showed significant modulations of G*f* (Santarnecchi et al., 2013, 2016) as well as with available neuroimaging literature addressing the localization of G*f*-related processes in the brain (Cole et al., 2012). The final selection of montage also overlapped with those used in the vast majority of the available literature on cognitive enhancement and tES (Santarnecchi et al., 2015).

The frequency spectrum of transcranial random noise stimulation (tRNS) used in this study was 100–500 Hz. This could be referred to as “high-frequency random noise” stimulation (tRNS applied in a frequency band below 100 Hz is commonly reported as “low frequency” tRNS). As documented in the original publication by Terney et al. (2008) and Santarnecchi et al. (2015), random noise electrical stimulation above 100 Hz seems to elicit a significant modulation of both electrophysiological and behavioral indices, with changes in cortico-spinal excitability measured *via* single-pulse transcranial magnetic stimulation (TMS), and a reduction of response times during a serial response time task. While low-frequency tRNS did not show any significant results in the work by Terney et al. (2008), additional significant effects have been reported by several groups using high-frequency tRNS, showing effects on motor learning (Cappelletti et al., 2013), perceptual learning (Fertonani et al., 2011) and arithmetic training (Snowball et al., 2013). Due to a limitation in the sampling rate of the brain stimulation device used for the present investigation (500 Hz), random noise stimulation was delivered between 100–500 Hz instead of a 100–640 Hz window used in the aforementioned publications. Given the frequency unspecific effects of tRNS, and its mechanism of action based on the injection of white noise and corresponding increase in the signal-to-noise ratio of the targeted brain region *via* stochastic resonance (Chaieb et al., 2011; Fertonani et al., 2011), we expected 100–500 Hz tRNS to produce the same or very similar effects as the original 100–640 Hz version.

The current density selected in this study was within the safety guidelines for tES reported by Neuroelectrics, though they differ from the values one expects from a “canonical” 5*7 cm electrode. The pi-stim electrodes used in this study differ from other electrode solutions usually applied in similar investigations, as they are based on sintered Ag/AgCl pellets and require conductive gel, rather than the more typical saline solution. This arrangement, though relatively novel, provides a more uniform current delivery. As for the canonical formula Intensity/Area, as suggested by the manufacturer^1^, the resulting current density does not increase linearly with electrode size, making a comparison with other publications using 5*7 or 3*5 sponge electrodes not entirely accurate (Miranda et al., 2009). From a more practical point of view, pi-stim has been used in several publications to achieve more focal stimulation solutions, with no ill effects being reported (Minhas et al., 2010; Borckardt et al., 2012; Faria et al., 2012; Murray et al., 2015). The same applies to our experience based on more than 900 stimulation sessions in more than 100 participants.

### Robot Factory

**Figure 1:**
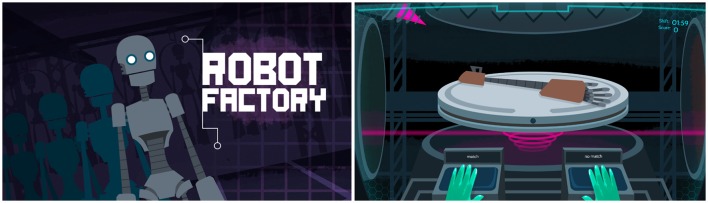
Robot Factory opening screen image (left), and example task screenshot from the Assembly Line scenario (right). In this task, participants are presented with robot arms one at a time on a moving platform, which rises from a portal at the bottom of the screen. Participants must decide which direction to sort the arm (indicated with the corresponding left or right “Shift” key), based on whether or not it is a match (in color and style) with the arm seen 2-previous. If the participant gives the correct response, the platform moves into the correct sorting tube and an icon appears indicating an increase in points. If the participant gives the incorrect response, the laser in the upper left corner shoots a beam onto the platform, dissolving the arm, and the platform recedes into the tube in the lower portion of the screen.

The behavioral portion of the FAST intervention is represented in the novel training game, Robot Factory (Figure 1; developed in collaboration with SimCoach Games^2^). Robot Factory was custom-designed based on the principles of the FAST framework and provides an engaging environment for completing a challenging and varied suite of EF tasks. In Robot Factory, participants are brought to a dystopian future in which they are employees of a factory producing robots. There they are asked to perform tasks related to building, sorting or programming ‘bots, according to instructions given every 2 min by their supervisor, Boss Bot. Task instructions are complex and reflect a “real world” application of one or more EF skills (e.g., programming a ‘bot by encoding a list of images as living beings/inanimate objects or flying/not-flying entities depending on cues presented in their work station during the shift). In Robot Factory, each 2-min “shift” represents a unique combination of EFs, logical or relational operators, stimulus domains, thematic context, stimuli and instructions. Progression through shifts is adaptive to a participant’s individual performance, such that task types and parameters for each session began at the level reached at the end of the previous session, keeping tasks challenging and requiring that participants maintain effortful performance.

In Robot Factory, participants are presented with two-alternative forced choice tasks, where they are asked to respond with either the Left Shift or Right Shift key. Shifts are composed of a variable number of trials, the duration of which could last 1, 2, or 3 s, depending on recent participant performance. In a participant’s first exposure to a task, stimuli were presented for up to 2 s, or until the participant gave a response. If a participant performed well on the task, stimulus durations on related tasks would be reduced to 1 s (or until the participant responded), but if the participant performed poorly on the task, stimulus durations would instead be extended to 3 s (or until the participant responded). A similar algorithm was implemented for the *n* of the Update *n*-back tasks, which varied between 1-, 2- and 3-back tasks. Feedback for each response was offered to the participant in both visual and auditory forms, and throughout training thematic music was presented to participants *via* headphones.

### Active Control

Our Active Control condition was created as a comparison group for our FAST intervention—one that is similarly challenging to FAST training, but which specifically does not target components of G*f*. By including an active control condition we address a significant limitation in the WM training literature, in which the results of training protocols are often compared only against a no-contact control condition (Buschkuehl and Jaeggi, 2010; Shipstead et al., 2010; Dougherty et al., 2016), and our design specifically and deliberately includes tasks that have been proposed by critics of WM training as candidate active control tasks (Redick et al., 2013). With a carefully-designed active control, we can be confident that improvements seen following our intervention are not simply a reflection of non-G*f* effects such as demand characteristics (Orne, 1962), low-level perceptual learning or Hawthorne effects (McCarney et al., 2007). Because interaction with experimenters has been shown to influence participant performance, our active control participants engaged in a similar overall experience to those in the intervention condition (same experimental setting, similar daily and overall training duration), which also controls for potential history effects (Shipstead et al., 2010).

In particular, our Active Control tasks were designed to recruit different cognitive functions and brain networks than those recruited by EFs or G*f* tasks—namely sensory and perceptual networks. In our efforts to maintain engagement levels in the Active Control group relative to the FAST training group, we incorporated a range of difficulty levels in each of the Active Control tasks, with trials ranging from very easy to very difficult. We also created a large set of tasks in an effort to allow for variety in the training, as a countermeasure against the relatively monotonous nature of the tasks. We expected performance on the control tasks to improve with training, but that improvements on these tasks would not generalize to G*f* task performance.

**Figure 2:**
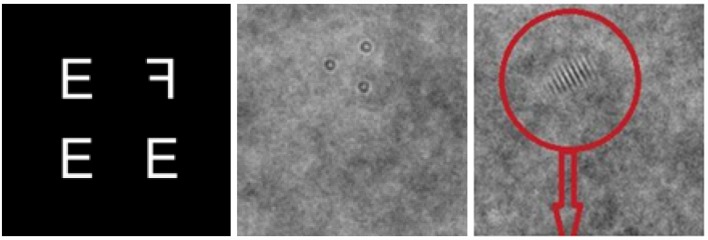
Example trials from the Adaptive Visual Search task (left), the Silo Detection task (middle) and the Gabor (“Fingerprint") detection task (right).

In the Active Control condition participants alternated between three, two-alternative forced choice tasks typically used to study low-level visual processing, implemented with adaptive difficulty (e.g., changes in the signal-to-noise ratio of stimuli; Figure 2): an Adaptive Visual Search task requiring the indication of whether a target letter “F" is facing to the left or the right [modeled after an active control condition recommended by critics of cognitive enhancement research (Redick et al., 2013)], a Silo Detection task requiring the identification of a triangular configuration of silos, and a Gabor “thumbprint" Detection task requiring the identification of a Gabor patch as being on the left or right half of a screen.

Similar to Robot Factory, in each task participants were asked to respond with either the Left Shift or Right Shift key, and task difficulty was manipulated based on participant performance. In the Adaptive Visual Search task there were 12 levels of difficulty based on: (i) the size of the letter-array in which the target letter was presented (2×2 to 12×12, in steps of 2); and (ii) whether the distractor letters were homogenous or selected from a random array of four letters (heterogeneous). Participants were moved to a higher level of difficulty if they achieved 87.5% accuracy or higher, and were moved to a lower level of difficulty if their performance dropped to 75% or below. In both the Silo and Gabor Detection tasks, difficulty was manipulated by changes to the signal-to-noise ratio (starting point: 0.5), with an incorrect trial resulting in an increase in stimulus coherence of 0.02, and two consecutive correct trials resulting in a decrease of coherence of 0.02. In all cases, participants had up to 3 s to respond, and trials terminated upon participant response. Difficulty levels at each session were based on the last level achieved on the previous day, and feedback was provided following each task block (all tasks: percent accuracy and average response latency; Silo and Gabor Detection tasks: latest task difficulty level). Throughout training, participants heard the same thematic music (*via* headphones) as that presented to participants in Robot Factory.

### G*f* Tests

Three standardized G*f* tests were used for our pre- and post-test assessment of G*f*: (1) the “advanced-short” version of the BOMAT, (2) Raven’s APM (Raven’s), and (3) a third matrix reasoning test with a large corpus of problems of varying difficulty adapted from Raven’s, developed by Sandia National Laboratories (Albuquerque, NM, USA), which we will henceforth term the Sandia test. The tests were performed in this order at both pre- and post-test for all participants.

Each test is made up of visual analogy problems in which a single item is missing from a complex matrix containing patterns varying in object shape, size, fill and orientation. For each test problem, the task is to select which of six (BOMAT) or eight (Raven’s, Sandia) possible response fits within the matrix. In the BOMAT and Raven’s tests, items increase in difficulty successively, whereas items of differing difficulty are randomly intermixed in the Sandia. Given constraints on the duration of pre- and post-tests, performance time for each G*f* test was limited to 15 min (45 min total for all three tests), an approach that has been taken in well-known previous work (Jaeggi et al., 2008). This duration is shorter than standard administration times for the BOMAT and Raven’s (typically 45 min each), thus further increasing the demands of the tests on our participants. Participants were informed of the time limit of each test, and were instructed to solve as many problems as they could during the duration, while also being accurate. Even with this constraint, our participants performed sufficiently well overall that some performed at or close to ceiling at pre-test for the Raven’s (with mean performance at pre-test lying 1.89 standard deviations below maximum score, as compared with 6.99 SDs for the BOMAT and 3.26 SDs for the Sandia).

### Bochumer Matrizen-Test (BOMAT)

The “advanced-short” version of BOMAT (Hossiep et al., 2001) is a nonverbal test of inductive and deductive reasoning, similar in style and structure to the widely used Raven’s APM (Raven’s). Like Raven’s, the advanced-short BOMAT is designed to differentiate those on the higher end of the G*f* scale and is particularly useful for testing highly intelligent individuals. Published A and B versions of the BOMAT were used for pre- and post-test, each of which consists of 29 items (one example and 28 test items of increasing difficulty). The BOMAT parallel forms were randomly counterbalanced across pre- and post-test sessions. BOMAT matrices are presented in a 3 × 5 format.

### Raven’s Advanced Progressive Matrices (Raven’s)

Raven’s APM (Raven and Court, 1998) is the most common test of nonverbal abstract reasoning ability. It is a nonverbal assessment that can be used to assess intellectual efficiency, high-level observation skills, clear thinking ability, and intellectual capacity. We divided the test into two parallel versions, each containing 17 test items of increasing difficulty, by approximating an even-odd distribution. The resulting distribution of item difficulty was similar for both versions. Raven’s matrices are presented in a 3 × 3 format.

### Sandia

Sandia matrices overcome the issue of a limited number of stimuli by providing the option to choose from a pool of approximately 3,000 matrices, obtained through the combination of different stimulus features like shape, color and orientation (Matzen et al., 2010). Experimental matrices belong to four different classes, based on the type and number of analogical operations required for a correct solution (1-, 2-, 3- relations, and logical matrices). Parallel versions of the test were created based on stimulus classes, which in our primary study contained 42 test items. Participants were limited to no more than 1 min on each test item given the relatively large n in the test. Sandia matrices are presented in a 3 × 3 format.

### Data Analysis

The overarching goal of our analysis was to determine whether participants’ G*f* ability improved as a result of our intervention (FAST+tRNS) compared to active (Active Control) and passive (No-contact) control conditions. G*f* ability in this study was measured by performance on three G*f* tests: BOMAT, Raven’s and Sandia. We calculated accuracy on these three tests at pre- and post-test (number of correct responses/total possible), and standardized those scores. We then created a measure of a single factor (G*f*) from those scores *via* confirmatory factor analysis, with Varimax (orthogonal) rotation and Bartlett’s weighted least-squares scores. Our factor analysis was constrained to a single factor, and explained a total of 49% of the variance in scores at pre-test, and 61.7% of the variance in scores at post-test (Table 1).

**Table 1:** Loadings and uniqueness (left), and sum of squared loadings and % total variance (right) for the G*f* factor at pre- and post-test.

	Loadings		Uniqueness				
	G*f* pre	G*f* post	G*f* pre	G*f* post		G*f* pre	G*f* post
BOMAT	0.476	0.672	0.773	0.549	SS loadings	1.469	1.852
Raven’s	0.497	0.748	0.753	0.44	% Total var	49	61.7
Sandia	0.997	0.918	0.005	0.158			

We then regressed the G*f* factor at post-test on both the G*f* factor at pre-test and Condition, based on the results of an Adjusted *R*^2^ model selection process (Table 2). The resulting β coefficients of our model represent the effect size of the FAST+tRNS training condition relative to each of the two control conditions.

**Table 2:** Model selection by Adjusted *R*^2^, for regression of post-test G*f* potentially controlling for G*f* score at pre-test, age and years of education.

AdjR2	(Intercept)	Gf_score_pre	Condition	Age	EduYears
0	X	-	-	-	-
0.633	X	X	-	-	-
0.639	X	X	X	-	-
0.637	X	X	X	X	-
0.635	X	X	X	X	X

To better understand the drivers of change in G*f* from pre- to post-test, we examined the correlation of progression in our training (FAST+tRNS) and Active Control conditions with changes in G*f*, as well as the relationship of performance in individual EF tasks and changes in G*f*.

## Results

### Examining Performance Across Suite of G*f* Tests

**Figure 3:**
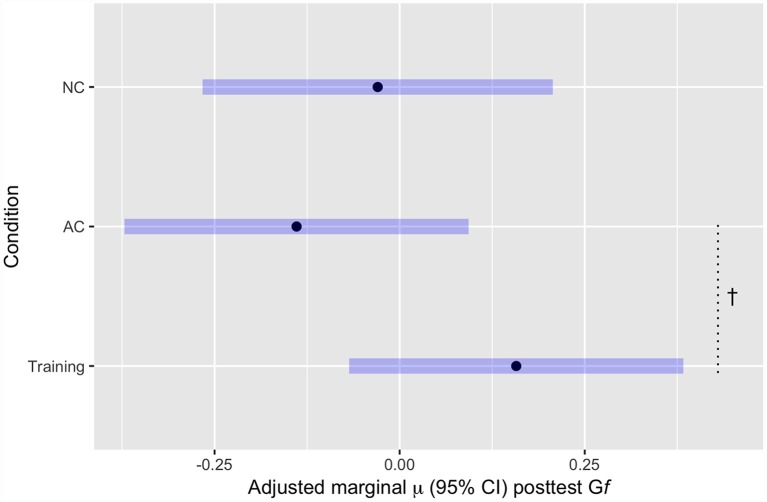
Mean G*f* post-test scores, adjusted for estimated baseline G*f* ability, by Condition. ^†^*p* < 0.1.

Our first set of analyses focused on the critical question of whether participants in the FAST-tRNS condition showed greater G*f* test performance at post-test relative to controls (Figure 3).

These results show greater predicted G*f* at post-test (controlling for pre-test G*f*) for the training group (FAST+tRNS) compared to both control groups, but neither difference reached statistical significance [FAST+tRNS vs. No-contact control: *β* = 0.187, 95% CI (−0.14, 0.51), *p* = 0.26; FAST+tRNS vs. Active Control: *β* = 0.3, 95% CI (−0.03, 0.62), *p* = 0.07; Figure 3]. No significant difference was found between the control conditions [No-Contact vs. Active Control, *β* = 0.11, 95% CI (−0.22, 0.44), *p* = 0.51].

### Examining Performance Within Each G*f* Test

**Figure 4:**
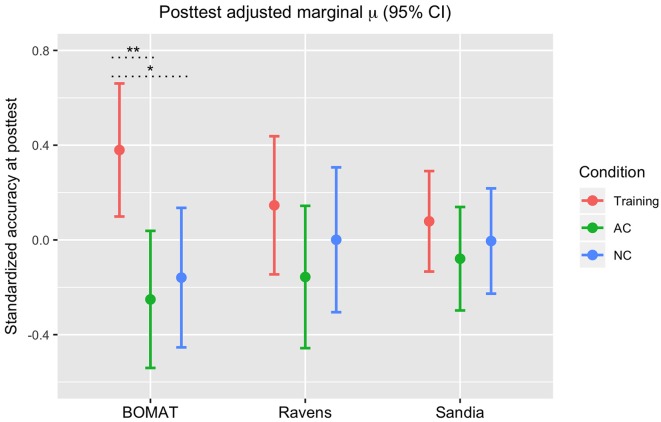
Standardized accuracy scores on each G*f* post-test (BOMAT, Raven’s, Sandia), adjusted for estimated baseline G*f* ability, by Condition. *p < 0.05, **p < 0.01, with Bonferroni correction.

Our study utilized three tests of G*f* as a means of better isolating a single latent factor (G*f*), but the results of our factor analysis indicated relatively high uniqueness of both our BOMAT and Raven’s tests relative to the Sandia (Table 1). Given this, our next analyses examined whether changes from pre- to post-test were differentially represented across our three G*f* tests. Here, we looked at standardized accuracy measures on each of the three G*f* tests, controlling for pre-test G*f* and Condition (Figure 4). A Bonferroni correction for multiple comparisons was used in these analyses.

*BOMAT*: we found significant differences in performance (standardized accuracy) between the FAST+tRNS training condition and both the No-contact [*β* = 0.54, 95% CI (0.13 0.946), *p* < 0.05] and Active Control conditions (*β* = 0.63, 95% CI (0.23, 1.03), *p* < 0.01), and no significant difference between the control conditions [*β* = 0.09, 95% CI (−0.32, 0.5), *p* = 0.66]. *Ravens*: we found no significant differences in performance (standardized accuracy) between the FAST+tRNS training condition and the No-contact [*β* = 0.15, 95% CI (−0.28, 0.57), *p* = 0.5] or Active Control conditions [*β* = 0.3, 95% CI (−0.12, 0.72), *p* = 0.16], and no significant difference between the control conditions [*β* = 0.16, 95% CI (−0.27, 0.59), *p* = 0.47]. *Sandia*: we found no significant differences in performance (standardized accuracy) between the FAST+tRNS training condition and the No-contact [β = 0.08, 95% CI (−0.22, 0.39), *p* = 0.6] or Active Control conditions [*β* = 0.16, 95% CI (−0.15, 0.46), *p* = 0.31], and no significant difference between the control conditions [*β* = 0.07, 95% CI (−0.24, 0.39), *p* = 0.64]. Altogether, we found numeric increases in post-test performance for the FAST+tRNS group in all three tests, but significant differences in performance only in the BOMAT.

### The Positive Impact of Training on Performance

Our next analysis focused on the FAST+tRNS group alone. If our FAST+tRNS training is effective at improving G*f*, then we can hypothesize that an individual’s degree of progression through the Robot Factory game should be predictive of the level of improvement in G*f* task performance from pre- to post-test. This is a strong prediction, since we might expect variation across participants in their level of motivation or effort to create a positive correlation with both pre-test score and FAST progression, acting against the predicted increase in post-test G*f* performance with training. To test this prediction, we ran a regression analysis of FAST+tRNS participants’ G*f* at post-test, as a function of progress in our training game, Robot Factory. Depending on whether FAST+tRNS participants completed 9, 10 or 11 days of training, they engaged in 135, 150 or 165 2-min “shifts" of Robot Factory tasks (15 shifts per day). Progress in Robot Factory was therefore defined as the proportion of those shifts “passed” by a participant, with accuracy >80%. Because Robot Factory was designed to be increasingly challenging, with more complex tasks occurring only after participants had mastered simpler tasks, a greater proportion of shifts passed meant faster progress through Robot Factory, to more difficult task types.

**Figure 5:**
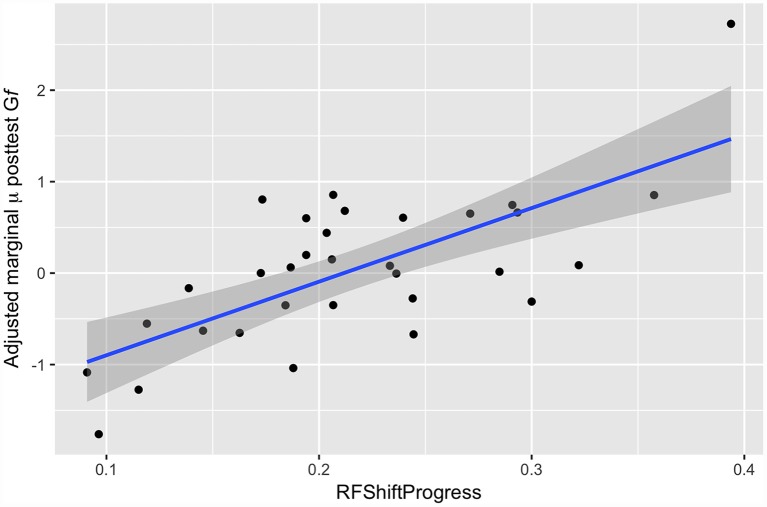
Adjusted marginal mean post-test G*f* as a function of progress in Robot Factory.

Here, we modeled post-test G*f* as a function of progress in Robot Factory (RF) and estimated baseline G*f* ability. Results of these analyses found a significant positive correlation between progress in Robot Factory and G*f* at post-test [*β* = 4.63, 95% CI (1.74 7.53), *p* < 0.001], with participants who progressed further through the FAST+tRNS training showing greater pre- to post-test change in G*f* (Figure 5).

### Changes in Executive Function Performance During Training

Given that Robot Factory is comprised of a series of tasks targeting EF practice, which serve as part of the foundation for improvements in G*f*, our final set of FAST+tRNS analyses examined changes in EF performance during training in Robot Factory. Because progression through Robot Factory (and corresponding task difficulty) was controlled by individual participant performance, a direct comparison of performance by day could not be made, as tasks on Day *n* for some participants might be significantly harder than tasks on the same day for other participants. Rather than comparing by training time point, our approach was to compare changes within individual participant performance, from the first $\frac{1}{3}$ of their trials in single executive function (single-EF) tasks to the last $\frac{1}{3}$ of their single-EF trials. With this metric, we can examine improvements in EF performance for each individual participant, which are relative to each individual’s progress.

**Figure 6:**
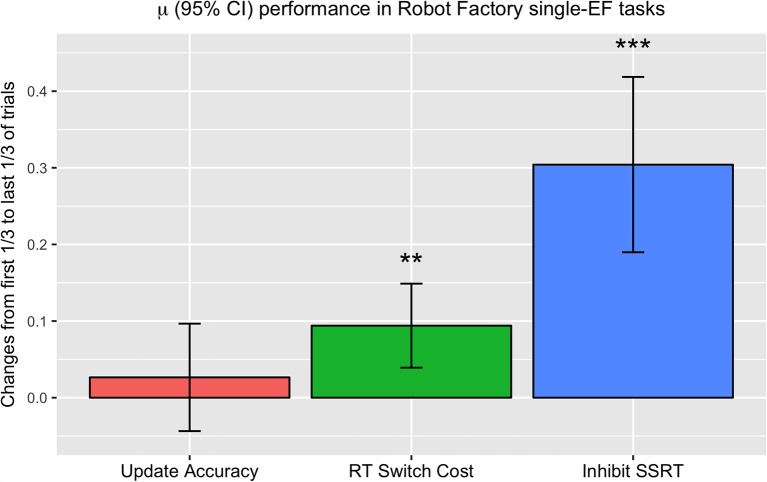
Changes in performance in the first $\frac{1}{3}$ of each participant’s trials in single-EF tasks to their last $\frac{1}{3}$ of trials in single-EF tasks. Measures are plotted so that positive values indicate improvement with training, giving the degree of accuracy improvement in Update trials (left), degree of decrease in average RT switch cost in Switch trials (middle), and degree of reduction in average stop-signal reaction time (SSRT) in Inhibit trials (right). **p < 0.01, ***p < 0.001.

For this analysis, we examined performance in single-EF tasks (excluding those tasks/shifts which combined multiple EFs) and compared the average of the first $\frac{1}{3}$ of trials for each participant with the average of the last $\frac{1}{3}$ of trials for each participant (Figure 6). We measured change in terms of improvement in *n*-back (update) task accuracy, reduction in switch costs, and decrease in estimated stop-signal reaction time (SSRT) in the stop-signal (inhibit) task (Logan and Cowan, 1984), such that positive values indicated improvement with training for all three measures. The results of this analysis found only a numerical increase in accuracy in the update task (*t*_(69)_ = 1.00, *p* = 0.16), and significant decreases in switch cost RT (*t*_(70)_ = 2.90, *p* < 0.01) and SSRT (*t*_(70)_ = 6.12, *p* < 0.0001).

If improvements in EF performance are part of the underlying mechanisms of G*f* improvement, then we would expect the changes we see in EF metrics to correlate with G*f* performance. In our final analyses, we examined G*f* at post-test, as a function of baseline G*f* and improvement in EF performance during training (individually added to separate models). From this we found no significant correlations among the individual tasks [*n*-back accuracy, *β* = 0.71, 95% CI (−0.41, 1.84), *p* = 0.21; decrease in switch cost RT, *β* = 0.3, 95% CI (−1.2, 1.78), *p* = 0.69; decrease in SSRT, *β* = 0.29, 95% CI (−0.41, 1.0), *p* = 0.4]. Overall, our analyses found that EF task performance within Robot Factory improved during training, but individual improvement in EFs did not significantly correlate with changes in G*f*.

### Does Progress in Active Control Tasks Also Lead to Improvements?

Finally, given that progress in Robot Factory strongly predicted G*f* gains from pre- to post-test, a reasonable question to ask is whether there was a similar impact of progress in our Active Control tasks on G*f* test performance. If so, it might suggest that the positive correlation between Robot Factory progress and G*f* gain reflects non-specific effects of adaptive training such as change in motivation. To answer this, we conducted a final analysis of only those participants who engaged in AC training, modeling their post-test G*f* as a function of progress in Active Control tasks, controlling for G*f* at pre-test. Here, progress in AC tasks was represented by the average difficulty level achieved on each of the three AC tasks on the last day of training (difficulty levels ranged throughout each training session), summed into a single score per participant. In these analyses, two participants had to be excluded based on recording errors in two or more of their AC tasks (final AC group, *n* = 28). Importantly, no significant correlation was found between progress in the Active Control tasks and G*f* post-test [*β* = −0.003, 95% CI (−0.06, 0.05), *p* = 0.91]: this negative-signed and very weak correlation between AC training progression and G*f* gain contrasts with the strong, positive correlation involving Robot Factory progression, providing reassurance that the latter reflects a meaningful, specific effect of this form of training on G*f* test performance.

## Discussion

The present study introduces a novel cognitive training intervention—Flexible, Adaptive, Synergistic Training (FAST)—that is designed to enhance G*f* through targeted, varied training of core EFs combined with brain stimulation. In the new Robot Factory game, EFs of working memory, cognitive flexibility, and inhibition are exercised in increasingly complex configurations and diverse task environments, reliant on dynamic task instructions. The FAST+tRNS intervention pairs this EF training with bifocal tRNS over DLPFC, a brain region that is a core component of a distributed fronto-parietal network strongly implicated in EF and G*f* (Duncan and Owen, 2000; Duncan et al., 2012). Together, this combination of EF training and targeted stimulation represents an approach that aims to target G*f* networks through synergistic activation by brain stimulation and EF practice, increasing activity during a critical state of enhanced plasticity. Our results suggest that the FAST+tRNS intervention may be effective in improving G*f* task performance compared against both no-contact controls, and, critically, against an active control group that engages in a similarly extended and challenging training regime targeting lower-level cognitive functions. Such improvements relative to an active control are fairly rare in the literature (Buschkuehl and Jaeggi, 2010; Shipstead et al., 2010; Dougherty et al., 2016), highlighting the importance of the comparison presented in this work. It should be noted that the central aim of the experiment described here was a feasibility study of a compound intervention consisting of a game and cortical stimulation—hence, the relative contribution of the individual elements of the intervention are difficult to establish and will have to be assessed in future confirmatory research.

The results of the study suggest transfer from the EF skills exercised in Robot Factory to matrix-reasoning of the G*f* tests. Our analyses showed improvements in EF task performance during training, as well as significant correlations of game progression and G*f* post-test performance. Our results also found numerically greater gains in G*f* for our FAST+tRNS training condition compared to both controls (Active Control, No-Contact Control), with a marginally significant difference between training and the Active Control Condition (no significant difference between training and No-Contact). Analysis of the individual G*f* tests found that these numerical gains were seen in all tests, but were driven most notably by significant increases in performance of the FAST+tRNS training group in the BOMAT, relative to both controls.

By comparing our FAST+tRNS intervention to a similarly-challenging training provided by the Active Control condition, we provide evidence against the interpretation that our training gains are the result of demand characteristics or a Hawthorne effect (Roethlisberger and Dickson, 1939; Sommer, 1968; Parsons, 1974). Furthermore, because our Active Control condition was carefully designed to emphasize low-level visual-spatial processing, the comparison of the FAST training-only and AC conditions effectively isolates the impact of our training on cognitive control processes (vs. increases in processing speed or visual search). These results offer initial evidence that visuospatial processing is not the main driver of improvements we see following our FAST intervention, though spatial working memory confounds resulting from the different matrix-sizes among the G*f* tests remain (Moody, 2009).

While correlation analyses indicate that improvements in G*f* task performance from pre- to post-test were strongly predicted by participants’ degree of progression through the FAST game (Robot Factory), progression in Active Control tasks was not correlated with such gains. This association is unlikely to reflect confounding effects of underlying G*f* ability or level of motivation, both of which could be expected to affect pre-test G*f* performance as much as post-test. Instead, the association can be understood in terms of the design of the Robot Factory training game, in which participants are given successively more complex combinations of EF training the further they progress. Progression through the game is determined by performance, so individuals who do well in simpler initial tasks are moved more quickly to more complex, higher-level tasks. This leaves these individuals more time to practice dual and even triple-EF tasks, as well as tasks with a dual *n*-back or hypothesis-testing component. These more difficult Robot Factory tasks require the most complex and structured organizations of component EFs and require them to be assembled rapidly with minimal instruction. We hypothesize that it is at these highest and most challenging levels of the game that participants are acquiring cognitive skills most relevant to G*f* task performance.

As this study served as an initial proof-of-concept for our training approach, additional work in this research program is currently underway tackling several important issues not specifically addressed in this study^3^. First, it is critical that the relative contributions of the FAST game (Robot Factory) and tES (here, tRNS) be more thoroughly explored. In this work, we cannot definitively say whether the gains we see are specifically the result of our training game alone, its combination with our stimulation protocol (or expectation effects induced by NIBS), or some combination thereof. A key aim of our future research will be to compare our FAST intervention with NIBS against a condition with Robot Factory training and sham stimulation, as well as to contemplate the effect of different forms and doses of NIBS. Nevertheless, these results do offer initial evidence for the possibility of enhancing G*f* through a short but intensive cognitive training regimen.

Regarding our training game, Robot Factory, a natural question is which of its many constituent components might be crucial to producing the observed G*f* gains: the requirement to combine EFs, to combine them in novel ways repeatedly over the course of training sessions adapted to participants’ abilities, to do so rapidly and with minimal verbal instruction, or to engage in hypothesis-testing to derive complex tasks from feedback. This issue can be addressed in future research by leveraging the complexity and richness of our training intervention to support an examination of relative contributions of the various elements of the training, provided the study has enough power (McKanna et al., under preparation).

Overall, the present research represents a potentially useful step forward in providing new insights into, and new methods for studying, the nature of G*f* and its malleability. Though our results await replication and extension, they provide preliminary evidence that while G*f* depends, as previously suggested, on core EFs of working memory, cognitive flexibility and inhibition, the crucial characteristic of G*f* may in fact be the ability to combine these functions rapidly and adaptively according to changing demand. More intriguing still, the ability to do so may be susceptible to targeted training that can lead to improvements in G*f*, with consequent implications for our understanding of the nature of intelligence as well as practical implications in light of the known predictive relationship between G*f* and a host of significant life outcomes.

## Concluding Remarks

While there has been widespread research interest in both characterizing the neural mechanisms underlying G*f*, and in developing effective and practical interventions, researchers have called for careful scrutiny and appropriate skepticism in assessing claims regarding the efficacy of interventions aimed at enhancing G*f*. These cautions are also relevant in the interpretation of the results presented here. The present study represents a preliminary test of a specific, theoretically-motivated hypothesis about activities that are likely to enhance G*f*. While results show promise, and reveal directions for future research, they do not form a sufficient basis for using the interventions described in clinical, educational, or personal enhancement contexts.

The study presented here reflects a limited set of performance measures, from a limited number of training conditions. The authors make no claims beyond the population of this study, but cite these preliminary results as evidence of the potential of enhancing G*f* through cognitive training. Future work will further explore the efficacy of FAST, comparing the impact of synergistic behavioral training + NIBS against training + sham stimulation, to determine the effects of expectation. Currently no plans exist for marketing of FAST or related trainings.

## Ethics Statement

In the US, these studies were approved by the institutional review board at all participating institutions (Harvard BIDMC: Committee on Clinical Investigations/IRB, Protocol 2014P-000024; Northeastern: Human Subject Research Protection/IRB, Protocol 14-08-15) and in the UK by the Berkshire National Research Ethics Committee (REC reference 14/SC/0131). All participants gave written informed consent prior to training, according to the Declaration of Helsinki, and were remunerated for their participation (£163; 12 and $11–20 per hour depending on site, in the UK and the US, respectively).

## Author Contributions

SM was the Principal Investigator and Program Manager of the SHARP Project. SM, AP-L, RC, MP and NY were responsible for the conceptualization of the SHARP Project, its funding acquisition and supervision. JA was responsible for the formal analysis, visualization and original draft writing of this manuscript. Review and editing of this manuscript was done by SM, JA, A-KB, FP, JM, ES, RC and NY. Investigations underlying this work were conducted by SM, JA, A-KB, FP, JM, ES, AP-L, RC, MP and NY, and the methodology employed here was co-determined by SM, JA, MP and NY.

## Conflict of Interest Statement

Prof. AP-L and Prof. RC serve on the scientific advisory board of Neuroelectrics Inc. Prof. AP-L also serves on the scientific advisory boards for Nexstim, Neuronix, Starlab Neuroscience, Axilum Robotics, Magstim Inc. and Neosync. These affiliations do not alter our adherence to Frontiers’ policies on sharing data and materials. Prof. AP-L is listed as an inventor on several issued and pending patents on the use of transcranial magnetic stimulation, and the real-time integration of transcranial magnetic stimulation with electroencephalography and magnetic resonance imaging Transcranial magnetic stimulation (tms) methods and apparatus (US 13/744,869; PCT/US2007/024694), Method and apparatus for recording an electroencephalogram during transcranial magnetic stimulation (US 09/067,111; US 09/746,055; PCT/US1999/008489), A method and a system for optimizing the configuration of multisite transcranial current stimulation and a computer readable medium and a computer program (US 14/058,517; PCT/IB2014/002180; EP20140793891), Identifying individual target sites for transcranial magnetic stimulation applications (US 14/401,296; PCT/US2013/032673), Method and apparatus for monitoring a magnetic resonance image during transcranial magnetic stimulation (US 09/096,725; PCT/US1999/013051). Prof. RC also led a patent entitled Apparatus for improving and/or maintaining numerical ability (US 13/578,125; PCT/GB2011/050211; EP20110702868). These patents do not alter our adherence to Frontiers’ policies on sharing data and materials. Listed authors SM and JA, and Honeywell SHARP Team authors Michael Dillard, and Umut Orhan are or were employed by the Honeywell Corporation at the time of authorship. Honeywell SHARP Team authors Garrett Kimball and Eben Myers are employed by the company Simcoach games. These commercial affiliations do not alter our adherence to Frontiers’ policies on sharing data and materials. The remaining authors declare that the research was conducted in the absence of any commercial or financial relationships that could be construed as a potential conflict of interest.

## Footnotes

^1^http://www.neuroelectrics.com/download/NE_UM_Part02_Electrode.pdf

^2^www.simcoachgames.com

^3^https://osf.io/26aun/
